# Metabolic gatekeepers: harnessing tumor-derived metabolites to optimize T cell-based immunotherapy efficacy in the tumor microenvironment

**DOI:** 10.1038/s41419-024-07122-6

**Published:** 2024-10-26

**Authors:** Yucheng Zheng, Rongwei Xu, Xu Chen, Ye Lu, Jiarong Zheng, Yunfan Lin, Pei Lin, Xinyuan Zhao, Li Cui

**Affiliations:** 1 Stomatological Hospital, School of Stomatology, Southern Medical University, Guangzhou, Guangdong China; 2 Department of Dentistry, The First Affiliated Hospital, Sun Yat-Sen University, Guangzhou, China; 3 School of Dentistry, University of California, Los Angeles, Los Angeles, CA USA

**Keywords:** Cancer microenvironment, Tumour immunology

## Abstract

The tumor microenvironment (TME) orchestrates a complex interplay between tumor cells and immune cells, crucially modulating the immune response. This review delves into the pivotal role of metabolic reprogramming in the TME, highlighting how tumor-derived metabolites influence T lymphocyte functionality and the efficacy of cancer immunotherapies. Focusing on the diverse roles of these metabolites, we examine how lactate, lipids, amino acids, and other biochemical signals act not only as metabolic byproducts but as regulatory agents that can suppress or potentiate T cell-mediated immunity. By integrating recent findings, we underscore the dual impact of these metabolites on enhancing tumor progression and inhibiting immune surveillance. Furthermore, we propose innovative therapeutic strategies that target metabolic pathways to restore immune function within the TME. The insights provided in this review pave the way for the development of metabolic interventions aimed at enhancing the success of immunotherapies in oncology, offering new hope for precision medicine in the treatment of cancer.

## Facts

- Abnormal tumor metabolism is a crucial hallmark of cancer, influencing disease progression and prognosis.
- Metabolites derived from tumor cells critically mediate immune cell function.
- Tumor cell-derived metabolites can reshape the tumor microenvironment, subsequently affecting the therapeutic outcomes of immunotherapy.

## Open questions

- How do specific tumor cell-derived metabolites modulate the anti-tumor immune response or create immune suppressive microenvironments, particularly across various types of cancer and at different stages of disease?
- Can specific tumor cell-derived metabolites be precisely targeted to selectively enhance the effector function of T cells and other immune cells within the tumor microenvironment?
- How do tumor cell-derived metabolites interact with immune checkpoint pathways, and can these interactions be exploited to enhance the efficacy of checkpoint inhibitors?

## Introduction

**Fig. 1:**
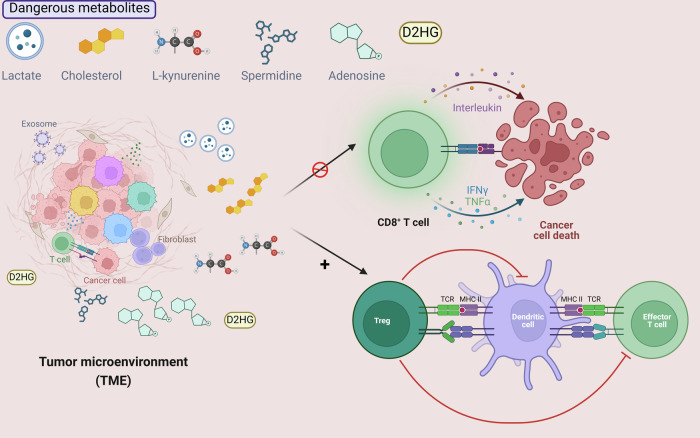
Tumor microenvironment and dangerous metabolites. The hypermetabolic tumor microenvironment (TME) generates a range of deleterious metabolites, including lactic acid, cholesterol, spermidine, l-kynurenines, and adenosine, which play a critical role in modulating the dual functions of immune cells in tumor progression and suppression.

The tumor microenvironment (TME) represents a highly complex ecosystem where tumor cells coexist with immune and non-immune cells, engaging in multifaceted interactions. Tumor cells are characterized by high metabolic rates, which lead to the production of various toxic metabolites that permeate the TME [1–4]. T lymphocytes, derived from the thymus, are crucial components of the adaptive immune response and play a pivotal role in tumor immunity. Within the TME, T cells exhibit a potent anti-tumor effect; however, they are also subject to the unique constraints imposed by this environment [5] (Fig. 1).

One prominent feature of tumor cell metabolism is the Warburg effect, where aerobic glycolysis leads to the accumulation of lactate. This accumulation decreases the pH of the surrounding environment, which can disrupt signal transduction involved in immune processes, thereby attenuating the anti-tumor capabilities of T cells [6, 7]. Furthermore, cancer cells’ altered lipid metabolism results in a high concentration of cholesterol within the TME. Elevated cholesterol levels can impair the cytotoxic activity of CD8^+^ T cells [8]. Notably, tumor cells also consume large quantities of amino acids, including glutamine, arginine, glycine, and serine. The metabolism of these amino acids generates a plethora of toxic byproducts [9, 10]. For instance, the metabolism of glutamine and tryptophan results in the production of γ-aminobutyric acid (GABA) and kynurenine, respectively. Both GABA and kynurenine have been shown to markedly inhibit the proliferation and anti-tumor activities of infiltrating CD8^+^ T cells [11, 12]. These metabolic interactions within the TME highlight the dual role of T cells: as key players in mounting an immune response against tumors and as victims of the suppressive conditions created by tumor cell-derived metabolites. Understanding these dynamics is essential for developing strategies to enhance the efficacy of immune-based therapies in oncology.

In recent years, there has been an increasing understanding of tumor cell-derived metabolites and their unique contributions to the TME’s complex ecosystem, as well as their roles in tumor progression and immune suppression. Our review systematically and critically summarizes recent findings on the impact of tumor cell-derived metabolites on T cell activities, providing a comprehensive resource for the field. Additionally, we detail the metabolic adaptations of tumor cells within the TME, offering insights into how these changes affect T cell function. Moreover, we discuss future research directions and therapeutic strategies that specifically target these metabolites to enhance the efficacy of immunotherapies.

## Overview of T cell generation and differentiation

**Fig. 2:**
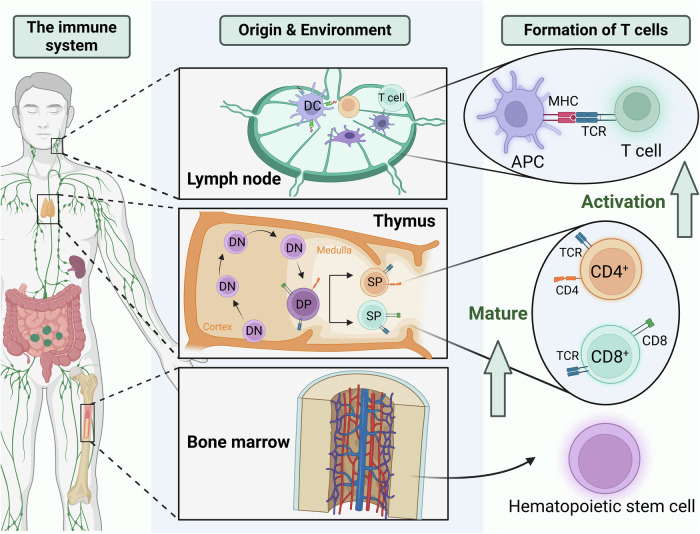
T lymphocyte maturation process. Hematopoietic stem cells in the bone marrow give rise to T lymphocytes, which migrate to the thymus for maturation. In the thymic cortex, DN T cell precursors, lacking CD4 and CD8 markers, undergo TCR gene rearrangement to generate a diverse TCR repertoire. They then progress to the DP stage, expressing both CD4 and CD8, and move to the thymic medulla for positive and negative selection. This selection ensures self-tolerance and functional competence, resulting in mature SP T cells. These mature T cells exit the thymus and populate peripheral lymphoid tissues, ready to respond to antigens presented by APCs via MHC–TCR interactions.

T cells originate from hematopoietic stem cells in the bone marrow and mature in the thymus before dispersing throughout the body via lymphatic and circulatory systems to populate immune organs and tissues [13, 14]. These cells facilitate adaptive cellular immunity by recognizing antigens presented on major histocompatibility complex (MHC) molecules on the surface of antigen-presenting cells (APCs) via T cell receptors (TCRs) (Fig. 2) [15]. This recognition triggers T cell activation, proliferation, and differentiation into effector cells that execute immune responses [16]. T cells are divided into two principal classes based on their differentiation markers and functions: CD4^+^ and CD8^+^ T cells. CD4^+^ T cells are activated by antigens presented by Class II MHC molecules, primarily developing into helper T cells (CD4^+^ Th), but they can also differentiate into regulatory T cells (Tregs). In contrast, CD8^+^ T cells are stimulated by antigens bound to Class I MHC molecules, differentiating into cytotoxic T lymphocytes (CD8^+^ CTLs) [17].

## Metabolic adaptations of tumor cells in the TME

**Fig. 3:**
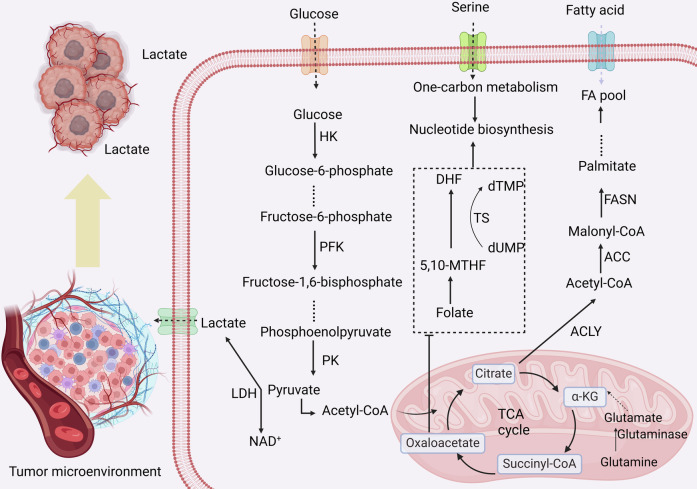
Metabolic processes in tumor cells. Cancer cells uptake glucose, fatty acids, and amino acids from the extracellular environment. Glucose is metabolized into lactate via glycolysis, which is excreted into the TME. Lipid metabolism is connected to the TCA cycle through the production of acetyl-CoA, which serves as a precursor for fatty acid synthesis. Amino acids, such as glutamine, contribute both to TCA cycle intermediates for energy production and to nitrogen metabolism, supporting various biosynthetic processes.

In the TME, the switch from oxidative phosphorylation to glycolysis, even in the presence of ample oxygen, is a hallmark of cancer metabolism [18]. This metabolic shift allows cancer cells to rapidly generate ATP and to divert glucose metabolites to anabolic pathways, facilitating biosynthesis, proliferation, and cell survival [19]. Critical enzymes in this pathway-hexokinase (HK), phosphofructokinase (PFK), pyruvate kinase (PK), and lactate dehydrogenase (LDH)-are highly overexpressed in many cancers [20–24] (Fig. 3). Notably, LDH facilitates the reduction of pyruvate to lactate, regenerating NAD^+^ required for glycolysis to continue rapidly. This production of lactate significantly acidifies the TME, impairing immune cell function [25].

Cancer cells reprogram lipid metabolism to support rapid cell division and to construct new cellular membranes [26]. Lipid reprogramming in cancer includes increased fatty acid synthesis, uptake, and altered lipid signaling, which together contribute to tumor growth and survival [27]. Key enzymes like acetyl-CoA carboxylase (ACC) and fatty acid synthase (FASN) are upregulated, driving de novo synthesis of fatty acids (Fig. 3). Additionally, the excessive production of cholesterol and fatty acids is not only crucial for membrane biogenesis but also modulates signaling pathways that regulate proliferation, apoptosis, and metastasis [28–32]. For instance, increased cholesterol levels suppress miR-33a and *SREBP2* mRNA expression, enhance PIM3 expression, and consequently stimulate CRC cell proliferation, accelerate cell cycle progression, and inhibit apoptosis [33]. Notably, cholesterol-rich lipid rafts serve as platforms for receptor-mediated signaling, enhancing oncogenic signaling pathways. Squalene epoxidase mediates cisplatin resistance through a cholesterol-dependent pathway that stabilizes c-Myc via lipid raft-localized Akt [34].

Cancer cells exhibit an increased demand for amino acids, not only for protein synthesis but also to support anabolic metabolism and redox balance [35]. Glutamine, in particular, is vital for cancer cells, serving multiple roles: it fuels the TCA cycle, supports nucleotide synthesis, and acts as a nitrogen donor for other biosynthetic processes [36–38]. Amino acids like serine and glycine are also pivotal, contributing to one-carbon metabolism, which is essential for nucleotide synthesis and methylation reactions necessary for DNA replication and repair [39]. Targeting amino acid metabolic pathways, therefore, presents a strategy to inhibit the supplies essential for tumor growth (Fig. 3). For instance, the glutamine antagonist prodrug DRP-104 significantly impairs the growth of KEAP1 mutant lung tumors by inhibiting glutamine-dependent nucleotide synthesis and enhancing anti-tumor T cell responses [40]. Similarly, glioblastoma excrete large amounts of branched-chain ketoacids (BCKAs) via monocarboxylate transporter 1. These excreted BCKAs suppress the phagocytic activity of tumor-associated macrophages, thereby contributing to tumor immune evasion [41].

The high rate of proliferation in cancer cells requires an ample supply of nucleotides for DNA and RNA synthesis [35]. Enhanced nucleotide metabolism ensures the availability of purines and pyrimidines, which are synthesized through both de novo and salvage pathways [42]. Given their critical role in DNA replication, enzymes involved in nucleotide synthesis are targeted by many chemotherapeutic agents, aiming to disrupt the proliferation of rapidly dividing tumor cells. For instance, targeting CSN6 decreases de novo nucleotide synthesis, increases chemosensitivity, and, when combined with butyrate treatment, improves chemotherapy efficacy [43]. Additionally, thymidylate synthase (TS) is intricately linked with EMT in cancer cells, showing elevated levels in mesenchymal-like cells. Targeting TS could reduce EMT traits and improve therapeutic outcomes [44] (Fig. 3).

## Metabolic influence of tumor cells on T cell dynamics

### The effects of tumor cell-derived lactate on T cells

In many cancers, tumor cells exhibit a high glycolytic rate, resulting in substantial lactate production and accumulation in the TME. This elevated lactate not only contributes to the acidic nature of the TME, but also has significant immunosuppressive effects on infiltrating T cells [45, 46]. Solute carrier family 16 member 3 (SLC16A3) encodes monocarboxylate transporter 4 (MCT4), responsible for exporting lactate and other monocarboxylates. High glycolytic activity in tumor cells leads to increased lactate production, and the transport of lactate mediated by SLC16A3 contributes to an immunosuppressive microenvironment, reducing the efficacy of immune checkpoint inhibitors by impairing CD8^+^ T cell function. Inhibiting SLC16A3 suppresses glycolysis and lactate efflux, thereby enhancing T cell-mediated tumor responses and improving outcomes with anti-PD-1 therapy. This identifies SLC16A3 as a promising target for overcoming resistance to immunotherapy in cancer [47]. Similarly, in glioblastoma, elevated lactate levels within the TME impede CD8^+^ T cell migration and infiltration, contributing to immune suppression. Through bulk and single-cell RNA-seq analyses, alongside machine learning and cell-cell interaction studies, these findings clarify how lactate modifies the immune landscape of glioblastoma [48]. Importantly, tumor-derived lactate impairs CD8^+^ T cell cytotoxicity by inhibiting pyruvate carboxylase and disrupting the TCA cycle, specifically anaplerosis. Targeting pyruvate dehydrogenase restores T cell metabolic functions, promoting succinate secretion and activating the succinate receptor, thereby maintaining T cell cytotoxicity in a lactate-rich TME [49]. This reveals pyruvate dehydrogenase as a critical therapeutic target to enhance T cell function in cancer treatment.

**Fig. 4:**
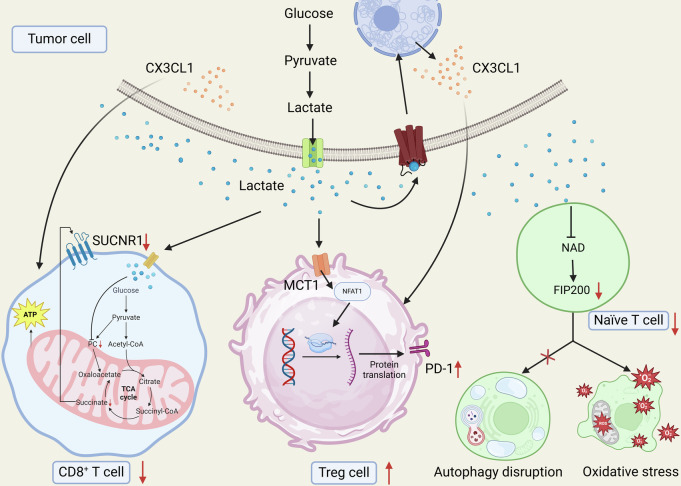
Effects of lactic acid produced by tumor cell metabolism on T lymphocytes. Lactate impairs CD8^+^ T cell cytotoxicity by inhibiting pyruvate carboxylase and disrupting the TCA cycle; MCT1 uptake of lactate enhances Treg cell function through NFAT1 nuclear translocation-mediated upregulation of PD-1 expression; and lactate induces apoptosis of naïve T cells by disrupting autophagy, mitochondrial hyperactivation, and increasing reactive oxygen species by inhibiting FIP200 expression.

In addition to CD8^+^ T cells, tumor cell-derived lactate also regulates the behaviors of other types of T cells in the TME. MCT1 is encoded by the *SLC16A1* gene and facilitates the transport of monocarboxylates like lactate and pyruvate across cell membranes, playing a key role in metabolic homeostasis and pH regulation [50]. Nuclear factor of activated T cell 1 (NFAT1) is a calcium-regulated transcription factor essential for T cell activation and function. It translocates to the nucleus upon calcium signaling, where it regulates genes involved in T cell differentiation, proliferation, and immune responses [51]. In glycolytic tumors, lactate accumulation in the TME promotes PD-1 expression on Treg cells more than on effector T cells, mediated by lactate uptake through MCT1 and subsequent NFAT1 nuclear translocation. This differential PD-1 upregulation enhances Treg cell function and may lead to PD-1 blockade therapy resistance [52]. Similarly, lactate in the TME promotes Treg infiltration via the G-protein-coupled receptor 81 (GPR81), enhancing immune tolerance in gastric cancer This lactate-mediated signaling involves upregulation of the chemokine CX3CL1, facilitating Treg migration and subsequently suppressing CD8^+^ T cell activity. Inhibition of GPR81 reduces Treg infiltration and impedes gastric cancer progression, highlighting lactate/GPR81 signaling as a target to counteract immune resistance in glycolytic tumors [53]. Notably, in ovarian cancer, tumor-derived lactate suppresses the expression of FAK family-interacting protein of 200 kDa (FIP200) in naïve T cells, leading to their apoptosis due to disrupted autophagy, mitochondrial overactivity, and increased reactive oxygen species. This metabolic targeting disrupts the balance between proapoptotic and antiapoptotic mechanisms in T cells, with consequences for anti-tumor immunity [54] (Fig. 4).

Emerging therapies that manipulate lactate metabolism are proving effective in enhancing T cell function and improving cancer immunotherapy (Table 1). For instance, lithium carbonate mitigates lactate-induced CD8^+^ T cell immunosuppression by blocking lysosomal acidification, which enhances lysosomal diacylglycerol–PKCθ signaling. This process promotes MCT1 localization to mitochondrial membranes, allowing lactate to be used as an energy source in T cells. These findings suggest that targeting lactate metabolism with lithium carbonate could enhance cancer immunotherapy by reprogramming T cell metabolic pathways to overcome immunosuppression [55]. Additionally, dichloroacetate mitigates the immunosuppressive effects of lactate in the TME by inhibiting macrophage ARG1 expression and the IL-23/IL-17 pathway, restoring CD8^+^ T cell and NK cell proliferation and function. Although dichloroacetate alone does not curb tumor growth, it enhances the efficacy of anti-tumor immunotherapy by correcting the metabolic disruption caused by lactate, underscoring its potential as an adjunct therapy in cancer treatment to boost immune response [56]. Similarly, dichloroacetate mitigates these effects by altering glucose metabolism in tumor cells, reducing lactate production. This metabolic reprogramming enhances T cell responses, increasing proliferation, cytokine output, and preventing apoptosis, thereby supporting anti-tumor immunity and potentially improving T cell-based therapies [57]. Dimethyl fumarate (DMF), an FDA-approved GAPDH inhibitor, counteracts the tumor-driven accumulation of lactate in the TME. By inhibiting glycolysis in tumor cells, DMF reduces lactate production, alleviating its suppressive effects on CD8^+^ T cells. This adjustment enhances T cell function and the efficacy of immunotherapies, presenting DMF as a viable strategy to modulate TME for improved anti-tumor immune responses [58]. The efficacy of IL-2 in T cell immunity is compromised by acidic tumor environments due to reduced STAT5 activation. A pH-selective IL-2 variant, Switch-2, has been engineered to activate CD8^+^ T cells more effectively in acidic conditions, enhancing anti-tumor responses and minimizing toxicity in normal tissues [59].

**Table 1:** Emerging therapies targeting lactate metabolism in cancer cells.

Compounds	Mechanisms	T cell functional effects	Impacts on tumor	Ref
Lithium	Block lysosomal acidification Rescue lysosomal diacylglycerol–PKCθ signaling Localize MCT1 to the mitochondrial membrane	Attenuate lactate-induced CD8^+^ T cell immunosuppression Improve T cell energy utilization	Improve tumor immunosuppression	[57]
Dichloroacetate	Inhibit macrophage ARG1 expression Inhibit IL-23/IL-17 pathway Reduce lactic acid production	Enhance T cell response	Promote anti-tumor immunotherapy	[58, 59]
Dimethyl fumarate	Inhibit GAPDH	Enhance TIL anti-tumor response	Inhibit tumor growth	[60]
Switch-2	Bind to IL-2 receptor subunit IL-2Rα Trigger STAT5 activation	Activate CD8^+^ T cells	Enhance anti-tumor response	[61]
Gd/CeO_2_	Oxidize lactic acid; generate -OH Induce mitochondrial damage	Activate CD8^+^ T cells	Enhance anti-tumor immunity	[62]
APAP-P-NO	Produce large quantities of nitrite S-nitrosylation impair GAPDH activity	Reduce Treg cells Promote CD8^+^ T cells infiltration	Reverse immunosuppression TME	[63]
HMONs@HCPT-BSA-PEI-CDM-PEG@siMCT-4	Inhibit lactate efflux	Restore T cell activity	Inhibit tumor growth and metastasis	[64]
Sodium lactate	Inhibit histone deacetylase in CD8^+^ T cells	Increase TCF-1 expression	Promote cancer immunotherapy	[65]

Interestingly, advanced materials have also been harnessed to rescue lactate metabolism, thereby reversing T cell suppression and boosting immunotherapy outcomes. For instance, material-engineered catalysts targeting intratumoral lactate enhance immunotherapy by catalytically depleting lactate, inducing metabolic reprogramming and apoptosis in tumor cells. This shift reduces glucose availability and induces mitochondrial damage, effectively activating M1-polarized macrophages and CD8^+^ T cells, thereby boosting anti-tumor immunity [60]. Interestingly, a novel tumor-specific peroxynitrite nanogenerator, APAP-P-NO, selectively disrupts metabolic homeostasis in melanoma cells, leading to significant changes in key metabolites. This disruption reduces lactate production and modifies the TCA cycle, effectively reversing the immunosuppressive TME. Consequently, there is an enhanced anti-tumor immune response, characterized by macrophage polarization, reduced suppressor cell populations, and restored CD8^+^ T cell infiltration, thereby increasing the efficacy of immunotherapies like anti-PD-L1 without causing systemic toxicities [61]. Importantly, a novel nanoplatform combining hydroxycamptothecin and siMCT-4 effectively targets lactate metabolism in tumor cells, inhibiting lactate efflux and inducing apoptosis. This intervention shifts tumor-associated macrophages from an M2 to an M1 phenotype, reactivating CD8^+^ T cell function and transforming an immunosuppressive TME into an immunoreactive one. This strategy enhances tumor immunotherapy, significantly reducing tumor growth and lung metastasis in vivo, and offers a potent approach to convert “cold” tumors into “hot” tumors [62].

Lactate seems to exhibit a dual role in promoting anti-tumor immunity. This paradox highlights the critical importance of precisely regulating lactate levels within the TME. While excessive lactate can inhibit immune responses, optimal levels can enhance the stemness and efficacy of CD8^+^ T cells through metabolic and epigenetic modifications. For instance, administration of sodium lactate in mice has been shown to increase the population of stem-like TCF-1-expressing CD8^+^ T cells within tumors, leading to significant inhibition of tumor growth. This finding suggests that a balanced approach to managing lactate concentrations could be crucial [63]. Reducing lactate excessively might diminish these beneficial effects on CD8^+^ T cell function, underscoring the need for precise modulation of lactate to harness its potential in enhancing anti-tumor immune responses. Thus, developing strategies that finely tune the lactate levels could provide a more effective means of controlling tumor progression and improving the outcomes of immunotherapies.

### The effects of tumor cell-derived lipid-related metabolites on T cells

**Fig. 5:**
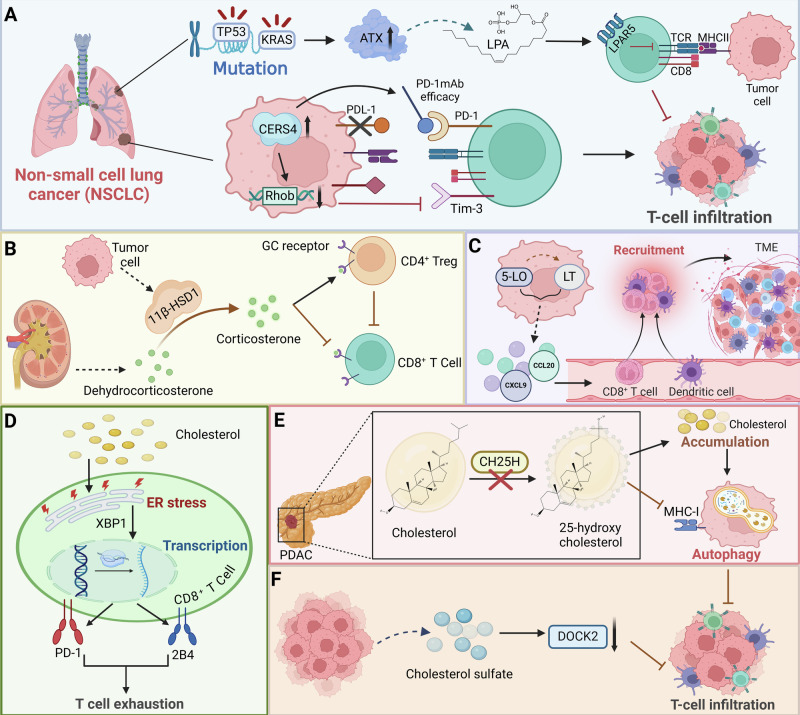
The effects of tumor cell-derived lipid-related metabolites on T cells. **A** In NSCLC, KRAS and TP53 mutant ATX and its metabolite LPA reduce CD8^+^ T cell infiltration, and the CERS4/Rhob/Tim-3 axis is strongly associated with T cell infiltration. **B** Tumor cells inhibit CD8^+^ T cell activation and promote Treg function through 11β-HSD1 activated glucocorticoids. **C** 5-LO and its metabolite leukotrienes recruit T cells and dendritic cells via the chemokines CCL20 and CXCL9 to mediates anti-tumor effects. **D** Tumor cell-derived cholesterol triggers CD8^+^ T cell depletion by increasing endoplasmic reticulum stress and activation of XBP1 to induce expression of immune checkpoints such as PD-1 and 2B4. **E** CH25H deficiency in PDAC causes cholesterol accumulation, promotes tumor progression, and reduces CD8^+^ T cell infiltration. **F** Cancer-derived cholesterol sulfate affects T cell infiltration by inhibiting DOCK2.

Tumor cell-derived lipid-related metabolites play a complex and critical role in shaping the immune landscape of the TME. These metabolites, including fatty acids, cholesterol, and other lipid intermediates, can profoundly influence T cell function and differentiation. For instance, in KRAS and TP53 mutant non-small cell lung cancer (NSCLC), the enzyme autotaxin (ATX) and its metabolite lysophosphatidic acid (LPA) suppress anti-tumor immunity by reducing CD8^+^ T cell infiltration. Elevated ATX correlates with lower CD8^+^ T cell activity and is linked to resistance against PD-1 inhibitors. Pharmacological inhibition of ATX or its receptor LPAR5, combined with anti-PD-1 treatment, restores T cell function and controls tumor growth [64]. Similarly, in NSCLC, the sphingolipid metabolizing enzyme CERS4 enhances anti-PD-1 therapy efficacy by influencing T cell ratios. Upregulation of CERS4 decreases Rhob expression, increases CD4^+^/CD8^+^ ratios, and reduces Tim-3^+^/CD8^+^ T cells, optimizing the immunotherapy response. Targeting the CERS4/Rhob/Tim-3 axis through sphingolipid metabolite regulation offers a promising approach to overcome resistance in NSCLC immunotherapy [65] (Fig. 5A). Additionally, tumor cells expressing the enzyme 11β-hydroxysteroid dehydrogenase type 1 (11β-HSD1) synthesize active glucocorticoids, which suppress CD8^+^ T cell activation and promote Treg functions, enhancing tumor progression. Disrupting 11β-HSD1, either genetically or pharmacologically, increases CD8^+^ T cell activity, decreases tumor growth, and improves response to PD-1 blockade [66] (Fig. 5B). Interestingly, 5-lipoxygenase (5-LO) and its metabolites leukotrienes play an unexpected antitumorigenic role by facilitating T cell and dendritic cell recruitment via chemokines CCL20 and CXCL9 (Fig. 5C). Mice deficient in 5-LO experience enhanced tumor growth and reduced immune cell infiltration in the TME, indicating that 5-LO products help regulate T cell function and suppress tumor progression. These findings caution against indiscriminate targeting of the 5-LO pathway with modulators like Zileuton and MK886 in lung cancer treatment strategies [67].

X-box binding protein (XBP) is a transcription factor crucial for the unfolded protein response. During endoplasmic reticulum (ER) stress, *XBP1* mRNA is spliced by inositol-requiring enzyme 1, producing the active XBP1s form, which regulates genes involved in restoring ER function and managing cellular stress [68]. Cholesterol in the TME triggers exhaustion in CD8^+^ T cells by inducing expression of immune checkpoints like PD-1 and 2B4, mediated through increased ER stress and activation of the XBP1 (Fig. 5D). Reducing cholesterol levels or inhibiting XBP1 in these T cells restores their anti-tumor activity, uncovering a metabolic mechanism for T cell exhaustion and presenting a novel approach to enhance the efficacy of T cell-based immunotherapies [8]. In addition, loss of cholesterol 25-hydroxylase (CH25H) in pancreatic ductal adenocarcinoma (PDAC) enhances cholesterol accumulation, promoting tumor progression and reducing CD8^+^ T cell infiltration by facilitating autophagy and downregulating MHC-I (Fig. 5E). Reintroduction of CH25H decreases PDAC cell viability under cholesterol scarcity and slows tumor growth, particularly when combined with immune checkpoint inhibitors, highlighting CH25H as a potential target for enhancing immunotherapy efficacy in PDAC [69]. Moreover, cancer-derived cholesterol sulfate inhibits DOCK2, a critical activator for T cell migration and activation, thereby preventing T cell infiltration into tumors [70] (Fig. 5F). Notably, a matrix metalloproteinase-2-sensitive nanovesicle is developed to enhance photodynamic cancer immunotherapy by targeting cholesterol metabolism in CD8^+^ T cells and tumor cells within the TME. This intervention reinvigorates T cell function and inhibits tumor cell migration, amplifying the immune response and significantly enhancing tumor growth suppression in a B16-F10 mouse model. This strategy offers a novel approach to augment cancer immunotherapy by manipulating cholesterol metabolism [71]. It should be pointed out that intratumoral T cells exhibit cholesterol deficiency, impairing their proliferation and promoting autophagy-mediated apoptosis [72]. Therefore, precise control of cholesterol levels in the TME becomes a potential strategy to optimize T cell functional states, preventing the exhaustion caused by over-activation and the functional suppression due to low cholesterol levels. Through pharmacological intervention or genetic editing techniques to precisely regulate cholesterol metabolism in the TME, it is possible to reactivate and enhance T cell anti-tumor responses, offering a new direction for cancer treatment. This approach must be carefully implemented to ensure that cholesterol levels are appropriately balanced, neither too high nor too low, to maximize the effectiveness of immunotherapy.

### The effects of tumor cell-derived amino acid-related metabolites on T cells

Tumor cell-derived metabolites such as spermidine, glutamate, and kynurenines result from the altered metabolism of amino acids, a process frequently upregulated to support rapid growth and survival under hypoxic conditions. By modulating local concentrations of these metabolites, tumor cells can exert profound immunomodulatory effects, such as inducing T cell exhaustion, impairing cytotoxic activity, and altering T cell metabolism. Understanding how these amino acid-related metabolites influence T cell behavior is crucial for developing strategies that can enhance T cell responsiveness and improve the outcomes of immunotherapies in cancer treatment.

**Fig. 6:**
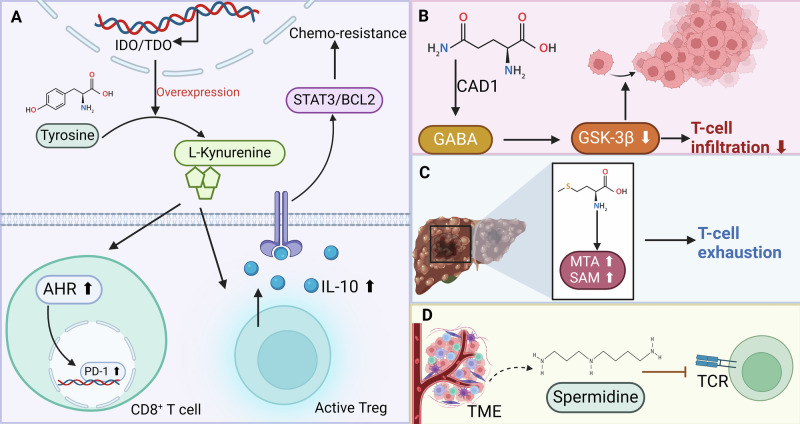
The effects of tumor cell-derived amino acid-related metabolites on T cells. **A** In tumors overexpressing IDO/TDO, the tryptophan metabolite l-kynurenine activates the AHR pathway, which enhances the expression of PD-1 on CD8^+^ T cells and activates Tregs leading to an increase in IL-10 secretion, thereby promoting chemoresistance via the STAT3/BCL2 pathway. **B** Cancer cells utilize glutamine to produce the neurotransmitter GABA via GAD1 and inhibit CD8^+^ T cell infiltration via β-catenin signaling. **C** Elevated levels of the methionine metabolites 5-methylthioadenosine and SAM are associated with T cell depletion in HCC. **D** Metabolite spermine from tumor cells inhibits TCR signaling.

In tumors overexpressing IDO/TDO, the tryptophan metabolite l-kynurenine activates the aryl hydrocarbon receptor (AHR) pathway, leading to increased immunosuppression via Tregs and tumor-associated macrophages, and enhanced PD-1 expression on CD8^+^ T cells. Inhibiting AHR, particularly alongside PD-1 blockade, counters this effect and slows tumor progression, suggesting a tailored immunotherapeutic strategy that combines AHR inhibition with checkpoint inhibitors to overcome resistance in IDO/TDO-high cancers [73]. Similarly, tumor-derived kynurenine hyperactivates Tregs, leading to increased IL-10 secretion which promotes chemotherapy resistance in gastric cancer via the STAT3/BCL2 signaling pathway (Fig. 6A). Targeting STAT3 not only disrupts this chemoresistance mechanism but also reduces growth and clonogenicity in tumor-derived organoid models, emphasizing the potential of inhibiting this pathway to overcome Treg-mediated chemoresistance in tumor settings [74]. Notably, IDO expression in melanoma cells is significantly lower than in mature dendritic cells, which excel in antigen presentation to CD8^+^ T cells. Tryptophan metabolites like l-kynurenine and 3-hydroxyanthranilic acid (3-HAA) do not affect antigen-specific CD8^+^ T cell responses. However, 3-HAA specifically inhibits the antigen-independent proliferation of CD8^+^ T cells driven by cytokines IL-2, IL-7, and IL-15, suggesting a selective impact of this metabolite on T cell homeostasis in non-antigenic conditions [75]. Additionally, cancer cells exploit glutamine to produce the neurotransmitter GABA via GAD1, impacting tumor biology and immune interactions. GABA activates the GABAB receptor, enhancing β-catenin signaling, which promotes tumor proliferation and inhibits CD8^+^ T cell infiltration [12] (Fig. 6B). Moreover, elevated levels of methionine metabolism products, 5-methylthioadenosine and *S*-adenosylmethionine (SAM), in HCC are linked to T cell exhaustion and poor patient survival (Fig. 6C). In vitro and mouse model studies show that disrupting SAM production via CRISPR-Cas9-mediated deletion of the MAT2A gene reduces T cell dysfunction and inhibits HCC growth, suggesting that targeting methionine metabolism could enhance anti-tumor immunity in HCC [76]. Importantly, spermidine, derived from tumor cells within the TME acts as an oncometabolite that suppresses TCR signaling by lowering plasma membrane cholesterol and inhibiting TCR clustering (Fig. 6D). Blocking polyamine synthesis, from which spermidine is produced, enhances CD8^+^ T cell-mediated anti-tumor responses, particularly when used in conjunction with anti-PD-1 therapy. This underscores spermidine’s role as a tumor-derived metabolic checkpoint in T cells, offering a novel target to improve the efficacy of tumor immunotherapies [77].

### The effects of tumor cell-derived adenosine on T cells

Adenosine in the TME is primarily sourced from the breakdown of ATP released by stressed or dying tumor cells and immune cells under hypoxic conditions. It impairs T cell proliferation and diminishes tumoricidal activities such as cytotoxicity and key cytokine production, mediated through A3 receptor interaction [78]. Similarly, the senescent TME, enriched in adenosine due to senescent tumor cells stimulating CD73 expression on tumor-associated macrophages via IL-6 and the JAK/STAT3 pathway, impedes anti-tumor immunity [79]. Moreover, blocking the CD39-adenosine pathway restores T cell proliferation and enhances CTL and NK cell cytotoxicity, highlighting a potential immunotherapeutic avenue to counteract tumor-mediated immune evasion [80]. Adenosine accumulation in solid tumors inhibits the motility of CD8^+^ T cells from HNSCC patients more than those from healthy donors, primarily through primarily through adenosine 2a receptor (A2aR) mediated suppression of KCa3.1 channel activity. A2aR signaling suppresses KCa3.1 channel activity by increasing cAMP levels, which in turn activates PKA, leading to the inhibition of KCa3.1. This reduces T cell motility since KCa3.1 is crucial for regulating ionic balance and membrane potential needed for migration. Activation of KCa3.1 channels with 1-EBIO restores T cell migration, overcoming adenosine’s inhibitory effects [81, 82].

**Fig. 7:**
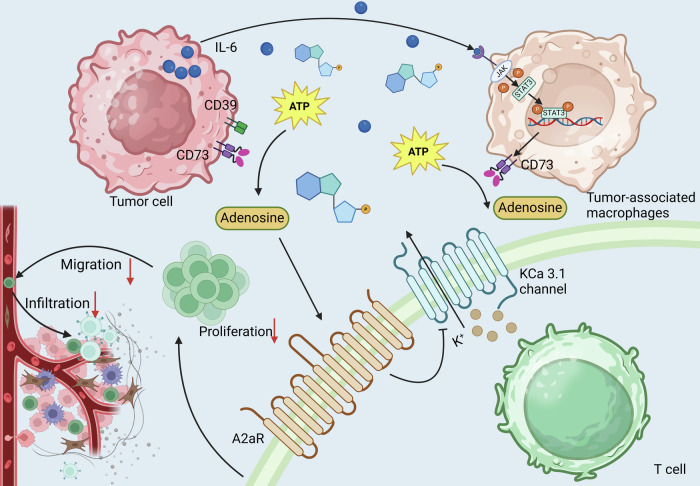
Effects of adenosine in TME on T cell function. Tumor cells with high self-expression of CD39 and CD73, which together convert ATP to adenosine, stimulate CD73 expression on TAM via the IL-6 and JAK/STAT3 pathways, which hydrolyze ATP to adenosine. Adenosine in the TME interferes with T cell proliferation, migration, and infiltration by activating A2aR and inhibiting its mediated KCa3.1 channel activity.

Genetic and pharmacological inhibition of A2aR enhances the anti-tumor activity of chimeric antigen receptor (CAR) T cells. Specifically, targeted knockdown of A2aR renders CAR-T cells resistant to adenosine’s inhibitory effects, and pharmacological blockade improves proliferation, cytokine production, and cytotoxicity [83]. Adenosine in the TME suppresses the function of mesothelin-specific CAR-T cells by activating A2aR [84, 85]. Silencing A2aR using siRNA-loaded nanoparticles in T cells from CT26 colon cancer models enhances anti-tumor activity by altering T cell metabolism. This is mediated through the downregulation of PKA, SHP2, and PP2Aα signaling pathways A2aR activation promotes PKA via cAMP signaling, leading to reduced glycolytic activity and increased Treg cell differentiation [86]. Meanwhile, SHP2 and PP2Aα contribute to inhibitory signaling, limiting glucose metabolism and effector T cell activation. Downregulation of these pathways reprograms T cell metabolism by promoting glycolysis, which provides the energy and biosynthetic precursors necessary for effector T cell function, enhancing cytokine production and driving an anti-tumor response [87]. Moreover, antagonizing the A2aR disrupts the hypoxia-adenosinergic regulation of T cells, promoting tumor rejection. Preladenant and its fluorinated analogs, developed through molecular modeling, were evaluated. Compound 29, a fluorinated triethylene glycol derivative, emerged as a potent modulator of T cell function in vitro, highlighting its therapeutic potential [88] (Fig. 7).

### The effects of other tumor cell-derived metabolites on T cells

Tumor-derived d-2-hydroxyglutarate (D2HG), an oncometabolite produced by mutant isocitrate dehydrogenase (IDH), impairs CD8^+^ T cell function by altering their metabolism through inhibition of LDH. This interaction reduces T cell cytotoxicity and IFN-γ signaling, affecting their anti-tumor efficacy. These findings, corroborated by clinical data from IDH1 mutant glioma patients, underscore d-2HG’s role in modulating immune responses in the TME, suggesting potential therapeutic targets for enhancing T cell activity in cancer [89]. Similarly, D2HG impairs CD8^+^ T cell and CAR-T cell function by inhibiting expansion and cytokine production. Modifying CAR-T cells with D2HGDH, an enzyme that converts D2HG to 2-oxoglutarate, enhances their anti-tumor activity. Overexpression of D2HGDH in CAR-T cells reduces serum D2HG levels, promotes T cell function, and improves survival in mice with IDH1-mutated NALM6 tumors, highlighting a potential strategy to enhance immunotherapy efficacy by metabolically reprogramming T cells [90].

As a crucial intermediate in the TCA cycle, succinate functions not only in metabolism but also as a signaling molecule that can modulate hypoxic responses in cells and is linked to inflammatory processes. Exposure to high succinate levels, often seen in SDH-deficient tumors like pheochromocytoma and paraganglioma, suppresses human CD4^+^ and CD8^+^ T cell activity by inhibiting cytokine secretion and degranulation. This effect is mediated by succinate’s accumulation and uptake via MCT1, which inhibits succinyl coenzyme A synthetase. This inhibition disrupts the TCA cycle, impairing glucose metabolism and consequently suppressing T cell function [91]. Chondroitin synthase 1 (CHSY1) is an enzyme crucial for the biosynthesis of chondroitin sulfate, a key component of the extracellular matrix. It functions by adding sugar residues to chondroitin sulfate chains, contributing to cell signaling, adhesion, and tissue structure [92]. CHSY1 exacerbates colorectal cancer metastatic progression by inducing CD8^+^ T cell exhaustion via the succinate metabolism pathway. Targeting CHSY1 with artemisinin, in conjunction with anti-PD-1 therapy, significantly reduces colorectal cancer liver metastasis. This dual approach, enhancing T cell function and inhibiting metastatic gene activity, offers a potent strategy for treating colorectal cancer liver metastases [93].

Increased levels of the tumor metabolite 5′-deoxy-5′-methylthioadenosine (MTA), stemming from a deficiency in the enzyme methylthioadenosine phosphorylase (MTAP), suppress T cell proliferation, activation, differentiation, and effector functions through modulation of the Akt pathway and interference with protein methylation. Conversely, in highly activated T cells, MTA exhibits cytotoxic effects. Restoration of MTAP expression in tumor cells enhances T cell proliferative responses, underscoring MTA’s role in tumor-induced immune evasion and its potential as a target for cancer immunotherapy [94]. Notably, PRMT5 inhibitors, such as EPZ015666 and naturally occurring MTA, reduce T cell proliferation, viability, and functionality, impacting essential cellular processes. Inhibition affects T cell metabolism and modulates key signaling pathways like p53 and AKT/mTOR. While targeting PRMT5 presents a therapeutic strategy for MTAP-deficient tumors, these findings highlight the potential immunosuppressive side effects on T cell responses, underscoring the need for careful evaluation of immune system impacts in cancer therapy development [95].

TET methylcytosine dioxygenase 2 (TET2) is an epigenetic regulator that catalyzes the oxidation of 5-methylcytosine in DNA, facilitating active DNA demethylation and influencing gene expression. It plays a critical role in cellular differentiation and metabolic regulation, including NAD^+^ metabolism [96]. Interestingly, lenvatinib enhances NAD^+^ metabolism in HCC cells by targeting the TET2 pathway, which increases NAD^+^ levels and induces tumor cell apoptosis. In patients with altered NAD^+^ dynamics, lenvatinib can restore NAD^+^ levels, counteracting the tumor’s metabolic adaptations that lead to immune evasion. For patients with elevated TET2 expression, lenvatinib’s ability to specifically target TET2 further enhances its impact on NAD^+^ metabolism and immune activation. This makes lenvatinib particularly beneficial for patients with low NAD^+^ levels or high TET2 expression, as it modulates both tumor metabolism and immune responses to inhibit tumor progression [97]. The effects of the above-mentioned tumor cell-derived metabolites on T cells were summarized in Table 2.

**Table 2:** The effects of other tumor cell-derived metabolites on T cells.

Metabolite	Mechanisms	T cell functional effects	Treatment	Ref
D-2-hydroxyglutarate	Inhibit LDH directly	Reduce cytotoxicity Impair IFN-γ signaling Inhibit T cell expansion Reduce cytokine production	D2HGDH-OE CAR-T cells catabolize and metabolize D2HG to enhance anti-tumor effects	[87, 88]
Succinate	Interfere with the TCA cycle Inhibit succinyl coenzyme A synthetase	Downregulate TNF and IFN-γ levels	Artemisinin targeting of CHSY1 significantly reduces liver metastasis in colorectal cancer	[80, 90]
5′-deoxy-5′-methylthioadenosine	Reduce regulation of Akt phosphorylation Interferon methylation	Decrease T cell proliferation, activation, differentiation and effector functions	Enhance PRMT5 activity to enhance T cell anti-tumor immunity	[91, 92]
Nicotinamide adenine dinucleotide	NAD^+^ participates in ATP synthesis Regulate cellular energy metabolism	Enhance T cell tumor removal function	Lenvatinib targets TET2 in HCC to promote NAD^+^ metabolism and enhance anti-tumor immunity	[93]

## Challenges and prospects

Understanding the complex interplay between tumor cell-derived metabolites and T cell function is pivotal for advancing cancer immunotherapy. These metabolites, varying from amino acids to lipids and nucleotides, can significantly alter T cell behavior, affecting their activation, proliferation, and effector functions. It is essential to delve into how these biochemical signals within the TME can suppress or enhance T cell responses. This insight not only sheds light on the intricate mechanisms of immune evasion but also opens new avenues for therapeutic interventions that can modulate these metabolic interactions to bolster T cell efficacy against cancer.

First, understanding the molecular mechanisms by which tumor cell-derived metabolites impact immune responses is crucial for enhancing the efficacy of cancer immunotherapies. The interaction between IL-7 signaling and adenosine-induced immunosuppression provides a clear example. IL-7 enhances the resistance of CD8^+^ T cells to the immunosuppressive effects of adenosine in solid tumors by promoting T cell accumulation and inhibiting FoxO1, a key transcription factor. By elucidating this pathway, new therapeutic strategies emerge that could simultaneously target the IL-7 and adenosine pathways, potentially reversing T cell suppression and improving cancer treatment outcomes [98]. Respiratory hyperoxia, where tissues are exposed to elevated oxygen levels, alters the TME by disrupting hypoxia, a key factor in tumor growth and immune evasion. This highlights the potential of manipulating environmental factors, such as oxygen, to influence metabolic pathways involved in tumor progression and immune suppression. Notably, by reducing hypoxia-induced adenosine accumulation, hyperoxia reverses A2aR-mediated suppression of immune responses, facilitating the activation and infiltration of tumor-reactive CD8^+^ T cells. This, in turn, increases pro-inflammatory cytokines and decreases immunosuppressive factors such as TGF-β, enhancing the effectiveness of immunotherapies. Therefore, a detailed understanding of how tumor-derived metabolites and conditions affect immune cells allows for the refinement of therapeutic strategies, ultimately leading to improved patient outcomes in cancer therapy [99, 100].

Second, the precise modulation of metabolite levels within the TME is a critical aspect of developing effective cancer immunotherapies. While it’s well-documented that several tumor-derived metabolites such as lactate and cholesterol can significantly suppress T cell-mediated anti-tumor activity, these metabolites also play essential roles in maintaining normal physiological functions of T cells. Therefore, indiscriminate reduction of these metabolites could paradoxically impair immune responses rather than enhance them, potentially leading to detrimental therapeutic outcomes. In light of these complexities, therapeutic strategies targeting the metabolic aspects of the TME must be finely tuned. For instance, interventions could be designed to selectively inhibit the metabolic pathways in tumor cells that produce immunosuppressive metabolites while sparing or even supporting the same pathways in T cells. Advanced drug delivery systems that target only tumor cells or specifically modulate the metabolite levels in the TME could provide one avenue for achieving this selectivity. Additionally, understanding the specific metabolic requirements and adaptations of different T cell subsets in the TME could lead to more precise interventions that support effector T cells’ functions while inhibiting Tregs or other immunosuppressive cells.

Third, although the impact of certain tumor-derived metabolites on T cell activity, such as lactate and adenosine, is well-established, the full spectrum of metabolites that regulate T cell function remains largely undefined. Numerous unidentified metabolites likely play significant roles within the TME, influencing T cell efficacy and overall immune response. Therefore, there is a critical need for comprehensive exploration and mapping of the tumor metabolome to uncover these hidden players. Advanced technologies like mass spectrometry and metabolomic profiling, coupled with high-throughput screening methods, could provide insights into complex metabolic interactions between tumor cells and T cells. By deciphering these intricate networks, novel therapeutic targets can be identified and strategies can be developed to manipulate these metabolites, thereby enhancing the effectiveness of immunotherapies.

Lastly, developing new metabolic antagonists and integrating nanotechnology in immunotherapies offer promising strategies to enhance CD8^+^ T cell anti-tumor activity by targeting specific metabolic pathways utilized by tumor cells. Metabolic antagonists such as IDO inhibitors can alleviate local immunosuppression, while nanocarriers can deliver these agents directly to the TME, ensuring targeted action and minimizing side effects. Combining these approaches with checkpoint inhibitors could further potentiate the immune response, creating a comprehensive treatment strategy. Ongoing research should focus on discovering novel targets through advanced genomic and proteomic studies and developing smarter nanotechnology-based delivery systems that respond dynamically to changes within the TME, paving the way for more effective and adaptive cancer immunotherapies.

## Conclusions

The intricate interplay between tumor cell-derived metabolites and T cell functionality in the TME underscores a significant yet complex landscape of immune modulation and metabolic interactions. These metabolites emerge not merely as byproducts of tumor metabolism but as potent regulators of both immune suppression and activation. Their profound impact on the efficacy of immunotherapies highlights crucial avenues for therapeutic intervention. Future strategies that target these metabolic pathways could transform the approach to immunotherapy, enhancing both precision and efficacy of cancer treatments. Achieving this will require a sophisticated understanding of the metabolic signatures unique to different tumor types and their corresponding immunological contexts. Integrating metabolic modulation with current immunotherapeutic strategies presents a promising frontier, potentially overcoming current limitations and pioneering a new era of metabolic engineering in cancer treatment.
